# 妊娠期噬血细胞性淋巴组织细胞增生症9例临床分析：特征、治疗策略与母婴结局

**DOI:** 10.3760/cma.j.cn121090-20250224-00092

**Published:** 2025-12

**Authors:** 燕平 刘, 祎 缪, 建勇 李

**Affiliations:** 南京医科大学第一附属医院，江苏省人民医院血液科淋巴瘤中心，南京 210029 Lymphoma Center, Department of Hematology, the First Affiliated Hospital of Nanjing Medical University, Jiangsu Province Hospital, Nanjing 210029, China

**Keywords:** 妊娠, 淋巴组织细胞增多症，噬血细胞性, 肝功能损伤, 治疗, Pregnancy, Lymphohistiocytosis, hemophagocytic, Hepatic dysfunction, Therapy

## Abstract

**目的:**

探讨妊娠期噬血细胞性淋巴组织细胞增生症（HLH）患者的临床特征和治疗结局。

**方法:**

回顾性分析2010年1月至2023年12月于江苏省人民医院就诊的9例妊娠期HLH患者的临床资料，总结其临床特点及治疗转归。

**结果:**

共纳入9例患者，妊娠早、中、晚期分别为1例（11.11％）、4例（44.44％）和4例（44.44％）；初次妊娠5例（55.56％），再次妊娠4例（44.44％）。6例（66.67％）患者以肝功能损伤为首诊表现。5例（55.56％）患者终止妊娠，其中2例（22.22％）妊娠晚期的患者胎儿存活、3例（33.33％）妊娠早、中期的患者胎儿死亡。7例（77.78％）接受糖皮质激素单药治疗，3例（33.33％）完全应答（CR）；1例（11.11％）患者采用环磷酰胺、长春新碱联合地塞米松方案化疗后CR；1例（11.11％）确诊后放弃治疗。中位随访5.0（0.6～103.7）个月，6例（66.67％）患者死亡，3例（33.33％）无病生存。

**结论:**

妊娠期HLH临床表现隐匿，肝功能异常为常见首发症状，早期诊断有助于改善患者预后。对于合并自身免疫性疾病及需要保障胎儿安全的妊娠期HLH患者，大剂量糖皮质激素可优先作为控制炎症风暴的治疗选择，终止妊娠时机需个体化决策。

## Abstract (en)

**Objective:**

This study aimed to investigate the clinical characteristics and therapeutic outcomes of hemophagocytic lymphohistiocytosis（HLH）during pregnancy.

**Methods:**

Clinical data, including disease features, treatment responses, and maternal-fetal outcomes, of nine pregnant patients diagnosed with HLH at Jiangsu Province Hospital from January 2010 to December 2023 were retrospectively analyzed.

**Results:**

Among the 9 patients, HLH onset occurred in the first, second, and third trimesters in 1（11.11％）, 4（44.44％）, and 4（44.44％）cases, respectively. Five patients（55.56％）were primigravida, whereas four（44.44％）were multiparous. Hepatic dysfunction is the initial manifestation in six（66.67％）patients. Pregnancy termination was performed in 5 cases（55.56％）, with fetal death observed in 3 cases（33.33％）during early/mid trimester and fetal survival in 2 cases（22.22％）during the third trimester. Seven patients（77.78％）received glucocorticoids alone, achieving a complete response（CR）in three cases（33.33％）. One patient（11.11％）attained a CR after cyclophosphamide, vincristine, and dexamethasone chemotherapy, whereas one（11.11％）declined treatment. The median follow-up was 5.0 months（range, 0.6–103.7 months）. Six patients（66.67％）died, whereas three（33.33％）remained disease-free.

**Conclusion:**

HLH during pregnancy is characterized by an insidious onset, with abnormal liver function being the primary presenting feature in most cases. Early and accurate diagnosis is crucial to initiate timely treatment and improve maternal survival. High-dose glucocorticoids are a prioritized treatment to control inflammatory storms in HLH during pregnancy, particularly for patients with autoimmune comorbidities or those requiring fetal protection. Individualized decision-making regarding the timing of pregnancy termination is crucial.

噬血细胞性淋巴组织细胞增生症（HLH）是一种罕见的遗传性或获得性免疫调节功能异常疾病，其特征是细胞毒性T淋巴细胞和巨噬细胞不受控制地激活，导致细胞因子介导的组织损伤和多器官功能障碍[1]–[2]。HLH分为原发性和继发性，原发性HLH是一种遗传性疾病，由影响细胞溶解功能、淋巴细胞存活或炎性小体激活的基因突变或基因变异引起，而继发性HLH主要由慢性炎症、感染或恶性肿瘤等因素导致[2]–[3]。HLH作为一种进展迅速的高致死性疾病，预后极差，未经治疗的患者中位生存时间不足2个月[4]–[6]，因此，早期诊断及治疗显得尤其重要。妊娠期HLH极其罕见，缺少特异的临床表现，容易延误诊断及治疗[7]–[10]。为了解妊娠期HLH的临床特征，本文回顾性分析江苏省人民医院收治的9例妊娠期HLH患者的临床资料，以期为临床早期诊断妊娠期HLH提供参考。

## 病例与方法

1. 病例：本研究为回顾性研究，收集2010年1月至2023年12月江苏省人民医院收治的9例妊娠期HLH患者的临床及实验室检查资料。本研究经江苏省人民医院伦理委员会批准（批件号：2023-SR-572）后开展，并经批准豁免知情同意。

2. 诊断标准及疗效评价：所有患者的诊断均符合HLH-2004标准，即至少符合以下8项特征中的5项：①发热：体温>38.5 °C，连续>7 d；②脾大；③血细胞减少≥两系（HGB<90 g/L、PLT<100×10^9^/L、中性粒细胞绝对计数<1.0×10^9^/L且非骨髓造血功能衰竭所致）；④高甘油三酯血症和（或）低纤维蛋白原血症（甘油三酯>3 mmol/L或高于同年龄的3个标准差，纤维蛋白原<1.5 g/L或低于同年龄的3个标准差）；⑤在骨髓、脾、肝或淋巴结中发现噬血现象；⑥NK细胞活性减低或缺失；⑦血清铁蛋白升高：铁蛋白≥500 µg/L；⑧可溶性白细胞介素‑2受体（sCD25）≥6 400 pg/ml或2 400 IU/ml。

所有患者参照《中国噬血细胞综合征诊断与治疗指南（2022年版）》[11]重新进行疗效评价，完全应答（CR）定义为HLH所有可量化症状和实验室标志物的正常化，包括sCD25、铁蛋白、甘油三酯、HGB、中性粒细胞绝对计数、PLT和ALT水平。部分应答（PR）定义为≥2项症状或实验室指标改善25％以上，个别指标需达到以下标准：①sCD25水平下降1/3以上；②铁蛋白和甘油三酯下降25％以上；③不输血情况下：中性粒细胞绝对计数<0.5×10^9^/L者，增加至原水平的2倍以上，且>0.5×10^9^/L，中性粒细胞绝对计数（0.5～2.0）×10^9^/L者，增加至原水平的2倍以上，且恢复至正常范围；④ALT>400 U/L的患者，需下降50％以上。无效（NR）定义为未达到CR或PR标准。

3. 随访：采用查阅临床资料或电话随访方式，随访时间截至2025年1月30日，中位随访时间为5.0（0.6～103.7）个月。

4. 统计学处理：采用SPSS 24.0软件进行数据分析，结果数据以*M*（范围）或例数（％）表示。

## 结果

1. 临床特征：共9例患者符合妊娠期HLH诊断标准。患者中位年龄为24（21～38）岁，起病至诊断的中位时间为22（12～44）d，中位妊娠周数24（8～37）周，妊娠早期1例（11.11％）、中期4例（44.44％）、晚期4例（44.44％）。初次妊娠5例（55.56％），再次妊娠4例（44.44％）；7例（77.78％）为单胎妊娠，2例（22.22％）为双胎妊娠。2例（22.22％）患者出现其他妊娠并发症，其中妊娠期肝内胆汁淤积症1例、妊娠期急性脂肪肝1例。5例（55.56％）患者病因明确，其中细菌感染2例、EB病毒（EBV）感染1例、干燥综合征（SS）1例、系统性红斑狼疮（SLE）1例；余4例（44.44％）病因不明。所有患者起病时均出现了持续发热，中位体温峰值为39.5（39～41）°C。脾大7例（77.78％），肝大3例（33.33％），3例（33.33％）患者同时出现肝脾肿大，2例（22.22％）存在多浆膜腔积液，1例（11.11％）水肿（表1）。6例（66.67％）患者以发热伴肝功能损伤为首诊表现，其中5例（55.56％）以黄疸为主要表现，伴转氨酶轻中度升高；1例（11.11％）仅转氨酶升高（表1）。

**表1:** 9例妊娠期噬血细胞性淋巴组织细胞增生症患者的基线特征

例号	年龄（岁）	妊娠周数（周）	发热	脾大	血细胞减少≥两系	高TG或低FIB血症	铁蛋白^a^（µg/L）	骨髓噬血现象	肝大	ALT（U/L）	AST（U/L）	TBil（µmol/L）
1	24	8	+	+	−	+	2 600	+	−	456.8	618.1	154.3
2	22	37	+	+	+	+	42 100	+	+	221.0	486.8	79.7
3	21	29	+	+	+	+	21 800	+	+	428.2	673.8	230.9
4	23	32	+	+	+	+	>1 500	+	+	235.4	886.3	189.4
5	38	17	+	+	−	+	9 585	+	−	729.0	804.3	14.7
6	29	29	+	+	−	+	8 637	+	−	206.3	434.5	25.3
7	34	22	+	−	+	+	4 956	+	−	24.4	94.3	15.6
8	31	25	+	−	+	+	>1 500	+	−	42.8	112.3	152.9
9	23	24	+	+	+	+	3 025	−	−	16.5	79.8	18.6

**注** TG：甘油三酯；FIB：纤维蛋白原；ALT：丙氨酸转氨酶；AST：天冬氨酸转氨酶；TBil：总胆红素；+：有；−：无；^a^因部分铁蛋白水平超过检测上限（1 500 µg/L）时未行稀释后再检测，报告为>1 500 µg/L

骨髓形态学检查发现，9例患者中有8例（88.89％）出现噬血细胞现象，6例（66.67％）患者出现两系或三系血细胞减少。1例（11.11％）患者诊断时全血EBV阳性（1.06×10^3^拷贝数/ml），所有患者诊断时巨细胞病毒均阴性。所有患者均出现了铁蛋白升高（≥500 µg/L）和高甘油三酯或低纤维蛋白原血症（表1）。

2. 治疗与转归：疾病发展过程中，5例（55.56％）患者终止妊娠，其中妊娠早期1例（11.11％）、中期2例（22.22％）、晚期2例（22.22％）（表2）。1例（11.11％）妊娠早期（8周）患者在疾病发展过程中胎儿死亡（不全流产），行清宫术终止妊娠后开始接受联合化疗（环磷酰胺800 mg/m^2^，第1、15天；长春新碱1.4 mg/m^2^，第1天；地塞米松8 mg，第1～5天）。1例（11.11％）妊娠中期（17周）患者在疾病发展过程中胎儿死亡（稽留流产），经治疗好转后行清宫术终止妊娠。另外1例妊娠中期（24周）患者因对治疗无应答，出现心功能不全，胎死宫内，经多学科会诊后行剖宫产术终止妊娠，娩出一死胎（出生体重800 g，Apgar评分1分钟0分），术后转入重症监护室治疗，后续患者治疗反应良好，最终好转出院。2例（22.22％）妊娠晚期患者因担忧反复高热影响胎儿，均在疾病急性期未治疗前行剖宫产术终止妊娠，胎儿均存活，其中早产儿2例（龙凤胎），足月儿1名。2例（22.22％）患者自然分娩，1例患者在疾病进展过程中分娩发动，顺产1名女婴（早产儿；出生体重2 kg；Apgar 评分：1分钟：8分，5分钟：9分）；另外1例患者HLH治疗缓解后，继续妊娠，后续胎儿发育良好，最终顺产1名男婴（足月儿；出生体重3 kg；Apgar评分：1分钟：10分，5分钟：10分）。

**表2:** 9例妊娠期噬血细胞性淋巴组织细胞增生症患者的治疗与结局

例号	起病至诊断时间（d）	病因	妊娠阶段	终止妊娠	终止妊娠阶段	治疗方案	疗效	生存结局		生存时间^c^（月）
								患者	胎/婴儿	
1	14	病因不明	早期	是	治疗前	CTX+VCR+DXM	CR	死亡	死亡	3.4
2	22	病因不明	晚期	是	治疗前	放弃治疗	/	死亡	存活	0.6
3	32	细菌感染^a^	晚期	否	/	MP	NR	死亡	存活	1.5
4	26	细菌感染^b^	晚期	是	治疗前	MP	NR	死亡	存活	6.5
5	20	SS	中期	是	治疗后	MP	CR	存活	死亡	90.5
6	12	病因不明	晚期	否	/	MP	CR	存活	存活	96.6
7	44	EBV感染	中期	否	/	MP	NR	死亡	死亡	1.5
8	31	病因不明	中期	否	/	DXM	NR	死亡	死亡	0.6
9	17	SLE	中期	是	治疗中	MP	CR	存活	死亡	103.7

**注** SS：干燥综合征；EBV：EB病毒；SLE：系统性红斑狼疮；CTX：环磷酰胺；VCR：长春新碱；DXM：地塞米松；MP：甲泼尼龙；CR：完全应答；NR：无效；^a^腐生葡萄球菌（骨髓培养）；^b^乳杆菌（骨髓培养）；^c^截至末次随访时间；/：不适用

HLH诊断后，7例（77.78％）患者单独使用糖皮质激素治疗，1例（11.11％）患者接受联合化疗，1例（11.11％）放弃治疗出院。4例（44.44％）患者经治疗后获得CR，其中糖皮质激素单药治疗3例，联合用药治疗1例。糖皮质激素单药治疗CR的3例患者中，2例合并风湿免疫性疾病（1例SS，1例SLE），1例病因不明。联合化疗CR的1例患者在缓解3个月后疾病复发合并严重肺部感染（考虑巨细胞病毒肺炎）导致呼吸衰竭死亡。4例（44.44％）NR的患者中，2例（22.22％）患者回当地医院继续治疗，后续随访2例患者因疾病进展而死亡。1例（11.11％）患者在疾病急性期终止妊娠，剖宫产术后因合并肝衰竭、多重感染及糖皮质激素治疗NR而死于HLH进展。1例（11.11％）在激素治疗过程中自然分娩，分娩后仍持续高热，激素治疗NR，临床医师建议使用HLH-2004方案治疗，家属拒绝化疗并要求出院，患者最后因HLH进展而死亡。

截至末次随访，3例（33.33％）患者存活，6例（66.67％）患者死亡，其中起病至诊断时间<22 d的4例患者中，3例无病生存至今（表2）。

## 讨论

妊娠期HLH起病隐匿，HLH临床表现通常为急性或亚急性（1～4周），主要特征为持续高热（体温>38.5°C）和淋巴造血器官肿大（淋巴结和肝脾肿大），部分患者可能有非特异性皮肤受累，表现为红斑疹、水肿、瘀点或紫癜[12]–[13]。由于HLH的罕见性、临床表现的多样性和非特异性，妊娠期HLH的早期诊断具有挑战性。本研究中，HLH的中位诊断时间为22（12～44）d，研究发现早期诊断的患者具有更好的疗效及预后。因此，充分了解妊娠期HLH的疾病特征，对临床上早期识别诊断妊娠期HLH，维护孕产妇的生命健康具有重要意义。

妊娠相关肝脏疾病在孕妇中的发生率达3％，是妊娠期肝功能障碍的最常见原因[14]。本研究发现9例患者中有6例（66.67％）患者以发热伴肝功能损伤为首诊表现，其中5例（55.56％）以黄疸为主要表现，伴转氨酶轻中度升高，且5例患者最后均因NR而死亡。既往研究中也有关于以肝功能损伤为首诊表现的妊娠期HLH的个案报道[15]–[16]。因此，在临床工作中，对于妊娠期出现肝功能损伤，合并持续发热且抗生素治疗效果不佳时，在排除妊娠期相关肝脏疾病后，应尽早考虑妊娠期HLH的可能，完善相关检查明确诊断，缩短诊断时间，改善患者预后。

妊娠期HLH的发病因素复杂，其可能由先天免疫反应或炎症性疾病驱动，还可能由遗传、恶性肿瘤、药物、感染（细菌、病毒、真菌）等多种因素组合驱动，甚至有些HLH患者的病因及感染病原体是未知的[17]。感染是继发性HLH的常见诱因，常见于EBV、疱疹病毒、利什曼原虫、流感病毒以及近年流行的SARS-CoV-2等[18]–[19]。本研究中，5例（55.56％）患者可明确病因（感染，SS，SLE）。既往研究结果显示，伴有自身免疫性疾病的妊娠期HLH患者对皮质类固醇和环孢素A反应良好[20]。本研究中2例合并风湿免疫性疾病的妊娠期HLH患者，对糖皮质激素治疗反应良好，最终均获得CR并无病生存至今。Dunn等[21]也报道了1例既往有成人Still病的妊娠期HLH患者对大剂量糖皮质激素单独治疗反应良好，持续CR并顺利完成妊娠及分娩。因此，对于妊娠期HLH患者，积极排查病因有助于选择治疗方式，对于合并风湿免疫性疾病的患者，优先选择糖皮质激素治疗有助于更好地保障胎儿的安全。此外，有研究报道患者的HLH会在第2次妊娠中期恶化，提示妊娠与HLH的发病机制相关[7]。总之，妊娠期HLH病因复杂，临床诊断应进行多学科诊疗，评估其病因，除排查细菌、病毒感染和肿瘤外，积极排查有无风湿免疫相关疾病对于指导治疗有重要意义。

妊娠期HLH患者的治疗及管理需要多学科参与，产科管理对于确保母亲和胎儿的安全至关重要。虽然终止妊娠是产科治疗的重要手段，但HLH本身并不是终止妊娠的指征。Teng等[8]报道了对类固醇治疗无反应的患者在终止妊娠后HLH治疗缓解的案例。本研究中，1例患者因治疗NR，经剖宫产终止妊娠后对治疗反应良好，并获得缓解。另外2例妊娠中期的患者对糖皮质激素治疗无反应，多学科讨论后建议终止妊娠，患者及其家属经充分考虑后决定暂不引产，最后2例患者均因治疗效果差而死亡。Abdelhay等[22]的系统回顾性分析结果显示，104例妊娠期HLH患者中，共有52例患者在HLH疾病急性期终止妊娠，其中21例（40.4％）的终止妊娠有助于疾病稳定或逆转。因此，对于难治性患者，终止妊娠或许可以改善患者对药物治疗的反应，改善患者预后。

根据HLH-1994方案，HLH的一线治疗方案为依托泊苷+类固醇激素（疗程为8周），由于依托泊苷可能对胎儿发育产生严重损害，对于胎儿发育未成熟的妊娠期HLH患者，糖皮质激素单药治疗HLH是最常见和相对安全的方法。本研究7例（77.78％）患者单独使用糖皮质激素治疗，3例（33.33％）患者获得CR并长期生存，其中1例患者经糖皮质激素治疗缓解后继续妊娠，胎儿发育正常，最终产下1名男婴，且在随访过程中婴儿的生长发育正常。既往也有多项研究报道了单独使用大剂量糖皮质激素治疗妊娠期HLH成功的案例[9],[21]。因此，对于需要保障胎儿安全的妊娠期HLH患者，糖皮质激素可以优先作为控制炎症风暴的治疗选择。但糖皮质激素单药治疗妊娠期HLH的缓解率较低，本研究中7例单独使用糖皮质激素治疗患者中只有3例（42.8％）获得CR。一项系统回顾性研究结果显示，使用HLH-94/04方案治疗的34例妊娠期HLH患者中，只有8例（23.53％）对治疗无反应，26例（76.47％）患者获得部分或完全缓解[22]。此外，Song等[20]的研究结果显示采用甲泼尼龙或静脉注射用人免疫球蛋白治疗的10例患者中仅有2例有效，而使用依托泊苷的6例患者均获得缓解。

综上所述，妊娠期HLH起病隐匿、进展迅速，临床表现兼具多样性及非特异性，当妊娠出现不明原因肝功能损伤、抗生素治疗无效的持续性高热时，应考虑到妊娠期HLH的可能，早期完善HLH相关检查，缩短诊断时间，尽早治疗有助于改善妊娠期HLH患者的预后。对于妊娠期HLH患者，可优先选择大剂量糖皮质激素单药治疗，但当患者糖皮质激素治疗NR时，结合产科意见、患者及家属意愿，及时终止妊娠、加用依托泊苷或许有助于改善患者预后。由于本研究病例较少，后续仍需多中心大样本研究进一步验证。
